# Gut microbiota modulation: a novel strategy for prevention and treatment of colorectal cancer

**DOI:** 10.1038/s41388-020-1341-1

**Published:** 2020-06-08

**Authors:** Winnie Fong, Qing Li, Jun Yu

**Affiliations:** 1 Institute of Digestive Disease and Department of Medicine and Therapeutics, State Key Laboratory of Digestive Disease, Li Ka Shing Institute of Health Sciences, CUHK Shenzhen Research Institute, The Chinese University of Hong Kong, Hong Kong, Hong Kong; 2 School of Pharmacy, The Chinese University of Hong Kong, Hong Kong, Hong Kong

**Keywords:** Cancer prevention, Colorectal cancer

## Abstract

Research about the role of gut microbiome in colorectal cancer (CRC) is a newly emerging field of study. Gut microbiota modulation, with the aim to reverse established microbial dysbiosis, is a novel strategy for prevention and treatment of CRC. Different strategies including probiotics, prebiotics, postbiotics, antibiotics, and fecal microbiota transplantation (FMT) have been employed. Although these strategies show promising results, mechanistically by correcting microbiota composition, modulating innate immune system, enhancing gut barrier function, preventing pathogen colonization and exerting selective cytotoxicity against tumor cells, it should be noted that they are accompanied by risks and controversies that can potentially introduce clinical complications. During bench-to-bedside translation, evaluation of risk-and-benefit ratio, as well as patient selection, should be carefully performed. In view of the individualized host response to gut microbiome intervention, developing personalized microbiome therapy may be the key to successful clinical treatment.

## Introduction

Colorectal cancer (CRC) is the third most commonly diagnosed cancer and the second leading cause of cancer death, accounting for 1.8 million new cases and 881,000 deaths worldwide in 2018 [1]. Although population-based colonoscopy screening and treatment advances lower CRC incidence and mortality in some highly developed countries, a rising trend of incidence and mortality is still observed in a number of developing countries [2]. CRC arises from the accumulation of multifactorial perturbations involving genetic, epigenetic and environmental aspects. Particularly, environmental factors including dietary consumption of food carcinogens, physical inactivity, and cigarette smoking are known to play the most important role in CRC initiation and progression [3].

To understand the environmental influence on CRC, the gut microbiome is a newly emerging yet important field of study. The gut microbiota, which harbors about 100 trillion microbial cells, is a complex community of bacteria, fungi, protozoa, and viruses [4]. With the technological breakthrough of high-throughput microbiome sequencing, a comprehensive yet culture-independent microbial profiling become possible, which further enables scientists to establish functional linkage between the gut microbiome, host physiology, metabolism, immunity, and malignancy [5].

Often referred as the “forgotten organ”, commensal homeostasis of gut microbiota plays an important role in the host’s health. In recent years, accumulating evidence has suggested the causal relationship between intestinal microbial dysbiosis and CRC pathogenesis. Enrichment of several bacterial species in gut, including *Fusobacterium nucleatum*, *Peptostreptococcus anaerobius* and enterotoxigenic *Bacteroides fragilis*, have been identified to contribute to colorectal carcinogenesis by inducing tumor proliferation [6, 7], promoting inflammation [8], causing DNA damage [9] and protecting tumor from immune attack [7]. On the other hand, some bacteria, mostly probiotics, such as *Lachnospiraceae* species, *Bifidobacterium animalis* and *Streptococcus thermophilus*, are found to be depleted in CRC patients [10, 11]. These bacteria are suggested to exert a protective effect against CRC. Recent research also reported the substantial influence of commensal microbes in prognosis of cancer patients. Abundance of *Fusobacterium nucleatum*, the notorious pro-tumorigenic gut bacteria, was associated with shorter survival in a large-cohort patient study [12], while later functional investigations have unraveled its role in promoting chemoresistance in CRC patients by activating autophagy [13], which consequently leads to treatment failure or disease recurrence.

With the increasing knowledge of how gut microbiome contributes to carcinogenesis and affects treatment outcomes, gut microbiota modulation, aiming to restore gut microbial homeostasis, becomes a potential strategy for CRC prevention and treatment. Here we summarize different strategies of gut microbiota modulation, including probiotics, prebiotics, postbiotics, antibiotics, fecal microbiota transplantation (FMT), as well as their putative mechanisms of actions. On the other hand, we would also like to address the associated risks and controversies regarding these strategies, particularly some of these strategies are commonly deemed to possess an excellent safety profile. At the very end, some updates about their bench-to-bedside translation and their therapeutic implications in clinical CRC management are discussed.

## Strategies of gut microbiota modulation

### Probiotics

**Fig. 1:**
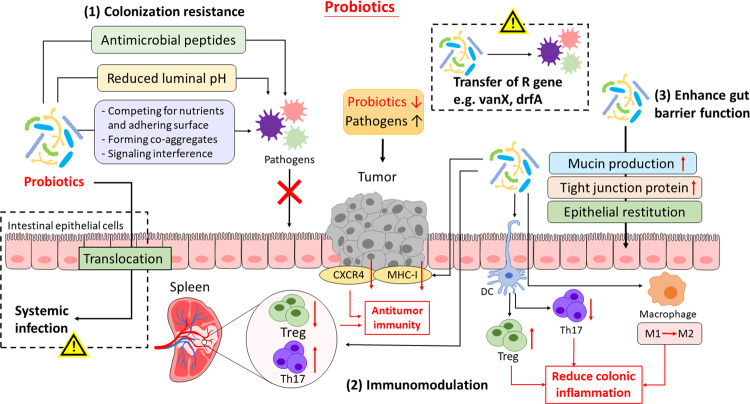
Putative mechanisms of actions of probiotics and their associated risks. Probiotics may implicate in CRC prevention and treatment by functioning in three different mechanisms: (1) Colonization resistance. Probiotics inhibit colonization of pathogenic bacteria by releasing antimicrobial peptides, lowering luminal pH and/or directly interacting with pathogens (e.g., competing for nutrients and location, forming co-aggregates). (2) Modulating immunity. Probiotics can have distinct immunomodulatory effect to reduce colonic inflammation (e.g., activating DCs, reducing Th17, increasing Treg expression and shifting macrophage to M2 subtype) or enhance antitumor immunity (e.g., enhancing Th17 and reducing Treg expression at a systemic level, reducing tumor CXCR4 and MHC-1 expression), subject to the selected species and strains. (3) Enhanced gut barrier function. Probiotics increase mucin production and tight junction protein expression and promoted epithelial restitution. However, there has also been some safety concerns regarding probiotic use in cancer patients, including the risk of bacterial translocation and systemic invasion, as well as the potential transmission of resistant genes to resident microbiota and the rise of antimicrobial resistance. CXCR, CXC chemokine receptors 4; DCs, dendritic cells; MHC-1, major histocompatibility complex class I; Th17, T helper cell 17; Treg, T regulatory cell.

Probiotics are defined as living microorganisms that confer health benefits on the host when administered in adequate amounts [14]. First hypothesized by Nobel laureate Élie Metchnikoff in the early 1900s, probiotics were described to modify the gut microflora composition and replace “putrefactive” bacteria with beneficial microbes [15]. As our understanding toward probiotics evolves, probiotics are now recognized to function beyond mediating the microbiota, but also induce physiological and metabolic changes in the host. The putative mechanisms of probiotics are summarized below and in Fig. 1.

#### Colonization resistance for pathogenic bacteria

Probiotic administration is suggested to restore microbial dysbiosis and maintain intestinal microbial balance by occupying host tissue and preventing colonization of pathogenic bacteria. Various studies have reported that ingestion of specific probiotic strains diminishes colonization of pathogens, including *Clostridium difficile* [16] and *Staphylococcus aureus* [17], thereby supporting the use of probiotics to prevent intestinal infection. Probiotics, or other commensal microbiota, confer colonization resistance by competing for nutrients [18] and adhering surface on epithelial cells or mucus [19], or alternatively by antagonizing pathogen colonization through aggregation with pathogens [20]. On top of direct interaction, probiotics can produce metabolites such as lactic and acetic acid, or bacteriocins, which inhibit pathogen growth by lowering luminal pH [21] and exert direct antimicrobial activity [22] respectively. A recent study has also reported the decolonization of *Staphylococcus aureus* by fengycins, an antifungal lipopeptide produced by the probiotic *Bacillus* species, via inhibiting quorum sensing, the bacterial signaling system [18]. By excluding pathogenic invasion, probiotic intake helps lowering risks of intestinal infection and subsequent inflammation, thereby potentially preventing CRC development, as well as reducing complications in preexisting CRC patients.

#### Mucosal immunomodulation

Probiotics exert an immunomodulatory effect in the gut and may (1) suppress colonic inflammation, or (2) enhance immunosurveillance, subject to the differential activity of each probiotic strain [23]. Through interacting with toll-like receptors (TLRs) and inducing retinoid acid metabolism, specific probiotic strains of *Bifidobacterium infantis* [24] and *Bifidobacterium breve* [25] are able to activate intestinal dendritic cells (DCs), leading to expression of Foxp3^+^ regulatory T cells (Treg) and type 1 regulatory T cells (Tr1) and IL-10 release. Some other probiotic bacteria, such as *Lactobacillus rhamnosus GG* and *Lactobacillus acidophilus*, downregulate the expression of Th17 cells and secretion of IL23 and IL17 via inhibition of STAT3 and NF-κB signaling [26, 27] or induce switch of macrophage phenotype, from pro-inflammatory M1 to immunosuppressive M2 [28].

On the other hand, probiotics may also work in a seemingly contradictory manner and induce a pro-inflammatory response. Probiotic-mediated activation of immune response, which involves the increased phagocytotic capacity and natural killer cell activity [29, 30], is traditionally implicated in eradication of infectious pathogens and potentiation of vaccine response. Yet in recent years, there are increasing interest toward its potential role in enhancing antitumor immunity. *Lactobacillus casei BL23*, a pro-inflammatory probiotic strain, has exhibited antitumor properties in dimethylhydrazine (DMH)-induced CRC mouse models. While downregulation of pro-inflammatory cytokines (MCP-1, TNF-α) and upregulation of IL-10 is observed in intestinal content, splenocyte analysis has demonstrated the decreased Treg level and increased Th17 population at the systemic level, thus triggering a Th17/Treg mixed-type immune response [31]. These results have suggested a fine-tuned regulation in anticancer immunity, putatively through the IL-2 signaling pathway [32]. Another probiotic strain, *Lactobacillus acidophilus NCFM*, was shown to suppress tumor growth in CT-26-implanted mouse models. The antitumor effect is postulated to stem from the reduced expression of CXCR4, which is implicated in outgrowth of micro-metastases, as well as the downregulation of MHC class I in tumor cells, resulting in subsequent T cell recognition and attack [33].

Of note, exhaustive efforts have now been devoted to characterizing specific cell-surface components responsible for the immunomodulatory effect, namely S-layer proteins, lipoteichoic acid and exopolysaccharides [34]. Through genetic modification or protein deletion, probiotics can be engineered to shift from a pro-inflammatory to an anti-inflammatory profile, or vice versa. For instance, deletion of lipoteichoic acid, the immunostimulatory protein, in Lactobacillus acidophilus downregulates expression of pro-inflammatory mediators and dampens colonic inflammation and CRC polyposis [35, 36]. These results have suggested probiotic engineering as an alternative strategy to attain the desired immunomodulatory effect.

#### Enhancement of gut barrier function

Gut barrier dysfunction, or increased tight junction permeability, has been a common feature in CRC [37]. A leaky gut, however, allows microbial translocation and promotes endotoxemia, leading to the development of cachexia [38]. The loss of tight junction protein in CRC is also implicated in induction of epithelial-mesenchymal transition (EMT) and metastasis [39]. Several probiotic strains, including *Lactobacillus rhamnosus*, *Lactobacillus plantarum* and *Escherichia coli Nissle 1917* are shown to improve gut barrier function by upregulating or normalizing expression of tight junction proteins (claudin-1, occludin, ZO-1, ZO-2) [40, 41], stimulating mucin production [42, 43], suppressing inflammation and promoting epithelial restitution [44]. By restoring the epithelial integrity, probiotics may exert beneficial effects on CRC patients.

Indeed, preclinical studies have suggested various plausible mechanisms that may potentially confer therapeutic benefits to CRC patients by manipulating gut microbiota. Yet, owing to its nature of ingesting viable microorganism, probiotic use has drawn a lot of suspicion and concerns regarding its safety profile in clinical use (Fig. 1).

#### Probiotic use in disease conditions?

Probiotics are generally considered safe and well-tolerated for healthy subjects, yet its safety profile has been challenged in patients with underlying medical conditions. Probiotic translocation, which refers to the entry of viable bacteria into extraintestinal sites and the ensuing systemic or localized infections, is one of the biggest concerns. Although bacterial translocation occurs also in healthy subjects, bacteria is normally sequestered and removed in the mesenteric lymph nodes under an intact immunity system, therefore conferring no detrimental effects. Such physiological protection, however, may fail in patients with damaged intestinal barrier or compromised immunity – which are also the clinical features presented in cancer patients and render them one of the susceptible populations [45]. Indeed, various case reports of probiotic-associated bacteremia, fungemia, endocarditis, liver abscess and pneumonia have been published [46], even though the ingested probiotics are known to possess low-virulent and non-pathogenic properties.

Nevertheless, as reported in some meta-analysis in cancer patients, incidence of these life-threatening side effects is rare, and it remains inconclusive whether probiotic use is associated with increased risk of infectious complications [47]. Current evidence does not suggest an absolute contraindication on probiotic use in cancer patients, but further clinical studies are warranted to confirm therapeutic benefits of probiotics and balance risks and benefits in infection-susceptible patients.

#### Transfer of resistant genes

Another theoretical risk regarding long-term probiotic use is the possible transmission of antibiotic-resistant genes via horizontal gene transfer (HGT). HGT, referring to the dissemination of mobile genetic materials within and between species, engendering bacteria to obtain resistant determinants and enhance survival under selective pressure (e.g., antimicrobial therapy). Of note, being a densely populated niche, our gastrointestinal tract is regarded as a large reservoir that allows transfer of antibiotic-resistant traits to bacteria colonized in close proximity [48]. For instance, a metagenomic analysis has shown that tetracycline-resistant genes (TcR) is commonly shared by the gut microbiota and is exacerbated by injudicious antibiotic use [49], which therefore suggested the occurrence of HGT in gut microbiota.

Owing to unclear clinical relevance, there are limited studies regarding antibiotic resistance in non-pathogenic bacteria, let alone probiotics, which are conventionally perceived to confer health benefits. When studying antibiotic resistance genes in probiotics, one of the critical considerations is to distinguish intrinsic and acquired resistance [50]. Further, the latter should be classified into non-transmissible (e.g., random genetic mutation on chromosome genes) and transmissible resistance (e.g., resistant genes located on plasmids or transposons, and readily transferred through HGT). The last resistance type is more of a concern in probiotic-mediated gene transfer. In fact, studies have reported the presence of antibiotic-resistant genes in mobile genetic elements of several probiotic strains, such as vanX gene in *Lactobacillus plantarum*, drfA gene in *Lactococcus lactis* and *Streptococcus thermophilus*, which encodes for vancomycin and trimethoprim resistance [51]. Interestingly, another ubiquitous resistance gene, tet(W), is located in the chromosome, yet is still potentially transferrable, due to its flanking sequence between transposase -encoding and -targeting sequence [52].

As demonstrated by some preclinical studies, transfer of resistant genes from probiotics to pathogenic bacteria do occur in the gut microbial communities via plasmids or transposons, the mobile genetic elements. Two commonly reported transmissible genes include ermB and tetM, which encode for macrolide and tetracycline resistance respectively, are shown to transfer from *Lactobacillus* or *Streptococcus* probiotics to potential pathogens such *Enterococcus faecalis* and *Listeria monocytogenes* [53, 54], introducing new resistant elements into these pathogenic bacteria.

However currently, studies regarding probiotics’ resistant gene transfer remains highly restrained in preclinical models and many questions about its clinical significance and impacts are yet to be answered. It has been extremely challenging to prove the association between probiotic ingestion and resistance development, due to multiple potential confounding factors in clinical settings.

### Prebiotics

**Fig. 2:**
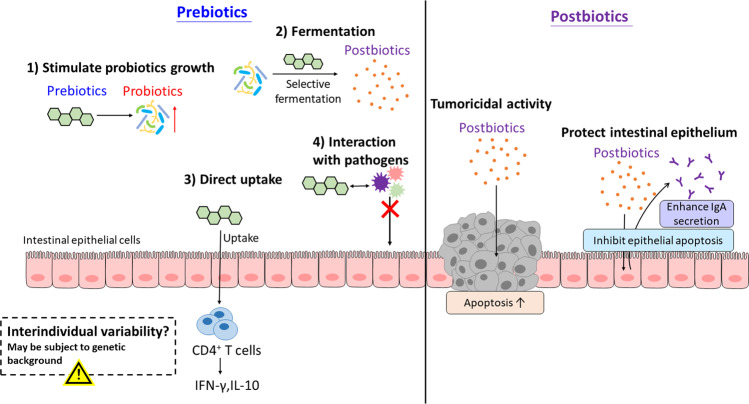
Putative mechanisms of action of prebiotics and postbiotics. Prebiotics function in the gut putatively via (1) stimulating probiotic growth, (2) selective fermentation by probiotics, (3) interacting with pathogens and preventing colonization and (4) being absorbed into intestine and exerting anti-inflammatory action, although the benefits of prebiotics may not be universal and subject to individual genetic background. On the other hand, postbiotics can (1) exert selective cytotoxicity against tumor cells and (2) protect intestinal epithelium by inhibiting apoptosis of normal epithelial cells and enhancing IgA secretion. IFN-γ, interferon-γ; IgA, immunoglobulin A; IL-10, interleukin-10.

The concept of prebiotics was first defined by Gibson and Roberfroid in 1995 as a nondigestible food ingredient that selectively stimulates the growth and/or activity of specific bacteria in the gut and improves host health [55]. Yet, later evidence has suggested a much broader scope of prebiotics, thus modifying its latest definition to “substrate that is selectively utilized by host microorganisms conferring a health benefit” in an expert consensus document in 2017. For example, while the term ‘nondigestible food ingredients’ only implies the conventional carbohydrate- and fiber-based prebiotics, other substances, such as the polyunsaturated fatty acids (PUFAs) and polyphenols, have been proposed to possess prebiotic potential over the last decade [56]. Furthermore, functionality studies on prebiotics have unraveled more complex actions beyond that of previously described (Fig. 2).

#### Modulation of gut microbiota composition

When prebiotics was first introduced, it was identified using culture-based models to evaluate its stimulation on specific probiotics, which were limited to *Lactobacillus* and *Bifidobacterium* species at that time [57]. Nevertheless, the recent advances in high-throughput sequencing technology have greatly expanded the scope. Several clinical trials reported the increased abundance of other putative probiotics, such as *Faecalibacterium* [58–60], *Akkermansia*, *Ruminococcus* and *Rosebura* species [60], after prebiotic administration. As discussed above, selective enrichment of probiotics in the gut is implicated in defense against pathogen and modulation of immune response. In various human studies, the decreased colonization of pathogens and dampening of inflammatory response, are observed in patients with chronic intestinal inflammation during prebiotic supplementation [61].

#### Production of fermentation metabolites

Prebiotics are selectively fermented by colonic probiotics, leading to the production of short-chained fatty acids (SCFAs) including acetate, propionate and butyrate. While butyrate is mainly taken up by colonocytes as major energy fuel, propionate and acetate are metabolized by liver and muscle for gluconeogenesis and energy generation respectively [62]. Functioning as a histone deacetylase inhibitor, butyrate has been suggested to exert beneficial effects on CRC patients by inducing CRC apoptosis, downregulating inflammation, modulating oxidative stress and enhancing epithelial barrier function, as reviewed elsewhere [63]. Propionate and acetate are much less characterized in the context of CRC or intestinal inflammation, but recent studies also reported the role of and propionate and acetate in suppressing colonic inflammation and protecting host against intestinal infection [64, 65].

#### Direct effect of prebiotics

In addition to stimulating probiotic growth and undergoing fermentation, prebiotics may act in a probiotic-independent manner and exert direct effect on the gut. One of the most studied directions is the antiadhesive properties against pathogens. By mimicking the microvillus glycoconjugates [66], prebiotic oligosaccharides can interact with the bacterial receptor and prevent pathogens from attaching to epithelial cells, thereby inhibiting pathogen colonization [67, 68]. Prebiotics are also postulated to be directly absorbed into intestinal cells and alter the gene expression profile. Using oligosaccharides with different degrees of polymerization (DP), a study has demonstrated that only prebiotics with low DP can enhance IFN-γ and IL-10 production in CD4^+^ T cells, which suggests its intact uptake through the intestine and subsequent modulation of intestinal immune response [69].

However, are these mechanisms going to universally benefit all subjects receiving prebiotics? Our current answer seems to give a no. Recent studies have unraveled an unexpectedly complex phenomenon – prebiotic interventions may exert variable effects in different individuals, and even more strikingly, may induce pernicious effects to host in some cases.

#### Interindividual variability in host response

Belcheva et al. have presented an interesting study that suggested the potential deleterious effect of prebiotic/ butyrate supplementation. In the study, the *APC*^*Min/+*^*;Msh2*^*−/−*^ mice were fed with low-carbohydrate diet or treated with broad-spectrum antibiotics, and both treatment groups were observed with attenuated polyp formation in small intestine and colon. Subsequent 16S rRNA sequencing has revealed a decreased abundance of butyrate-producing bacteria, namely *Clostridiaceae*, *Lachnospiraceae*, and *Ruminococcaceae*. Consistently, butyrate production is significantly reduced as found in the liquid chromatography-tandem mass spectrometry (LC/MS/MS) analysis. Collectively, these results seem to suggest the microbial-derived butyrate as an oncogenic metabolite that its depletion suppresses tumor development. For validation, butyrate was supplemented to the antibiotic-treated *APC*^*Min/+*^*;Msh2*^*−/−*^ mice. Remarkably, butyrate treatment has driven epithelial cell hyperproliferation, polyp formation, and eventually tumor progression [70]. These results are seemingly contradictory to numerous previous studies. Yet, an important point to be considered is the differences of host genetic background, which may plausibly explain the different tumor phenotype, oncogenic pathways and subsequently response to a specific intervention [71]. Therefore, prebiotic/ butyrate supplementation may not necessarily implicate health benefits to host, but individual variability does exist and is highly dependent on the somatic genetic background.

Echoing with Belcheva et al. studies, Singh et al. have also reported the detrimental microbial fermentation following prebiotic supplementation. The research team initially attempted to examine whether inulin mitigates metabolic syndrome in Toll-like receptor 5 (TLR5) knockout mice. Yet surprisingly, although long-term inulin-enriched diet does alleviate metabolic dysfunction, it promotes cholestasis and necroinflammation, and consequently induces hepatocellular carcinoma (HCC). Similar results were obtained for other soluble fibers (pectin and fructooligoscaccharide), but not in non-fermentable, insoluble fiber (cellulose). Further analysis revealed the enrichment of *Clostridia* species in mice that developed HCC, particularly Clostridium cluster XIVa, which is known to be the key producer of butyrate as well as the carcinogenic secondary bile acids. Depleting butyrate-producing bacteria reduced HCC incidence in TLR5 knockout mice. In addition, chronically supplementing inulin in drinking water induced hepatic inflammation and fibrosis but did not promote tumor development [72]. These have collectively suggested that prebiotic fermentation and butyrate production contribute partially to HCC development, although not being the decisive driver. More importantly, consistent with Belcheva et al. studies, such carcinogenic risk only occurs under a specific genetic background, further supporting the notion of interindividual variability in response to prebiotic intervention.

### Postbiotics

Postbiotics refer to the soluble byproducts and metabolites secreted by gut microbiota that exerts biological activities to the host. SCFA, produced from probiotic fermentation, is the most well-known example of postbiotics. For certain probiotic strains, it is the conditioned medium (or culture supernatants), instead of the viable bacteria, that exerts the desired effect. Therefore, postbiotics, in some cases, maybe an effective yet safer strategy when compared to ingestion of viable microorganisms [73]. Isolation and characterization of postbiotics, though still in its infancy, has thus attracted increasing interest in recent years. The putative mechanisms of some identified postbiotics are as follows (Fig. 2).

#### Protection of intestinal epithelium

Several postbiotics are postulated to suppress colonic inflammation and restore gut barrier integrity. A soluble protein derived from *Lactobacillus rhamnosus GG*, named p40, has been reported to inhibit cytokine-induced epithelial apoptosis, gut barrier disruption [42, 74] and enhance immunoglobulin A secretion [75] via transactivation of epidermal growth factor receptor (EGFR). Targeted delivery of hydrogel-coating p40 (to protect p40 from degradation) is effective in preventing and treating intestinal injury and inflammation, as well as promoting protective immune response [74]. Cell-free supernatant of several other probiotic strains, such as *Lactobacillus rhamnosus GG*, *Lactobacillus acidophilus*, *Lactobacillus casei* and *Bifidobacterium breve*, are also shown to downregulation inflammation or preserve gut barrier function primarily [76–78], though the exact identity of the postbiotics and the molecular mechanisms are not yet fully understood.

#### Selective cytotoxicity against tumor

Certain postbiotics, including lactate dehydrogenase or other unknown molecules from *Lactobacillus* species, have been shown to induce apoptosis or inhibit invasion in CRC cell lines [79, 80], yet most of these studies are highly limited by the lack of validation in in vivo models. A recent study has reported a potent tumoricidal effect of *Lactobacillus casei* ATCC334 supernatant, wherein ferrichrome is subsequently identified as the responsible molecule that induced apoptosis via JNK-DDTI3 signaling axis. The isolated postbiotic has exerted minimal effect on normal intestinal epithelial while having stronger antitumor activity than conventional CRC drugs [81], thereby suggesting the therapeutic potential of postbiotics.

Research about postbiotics is a rapidly growing yet highly unknown area. Owing to the substantial number and diversity of metabolites presented, it has been an enormous challenge for scientists to isolate the molecule responsible for the therapeutic effect, let alone to characterize its safety profile in preclinical and clinical settings. We will expect to see more safety information regarding postbiotics as the field is getting more sophisticated and developed.

### Antibiotics

#### Depletion of deleterious bacteria

**Fig. 3:**
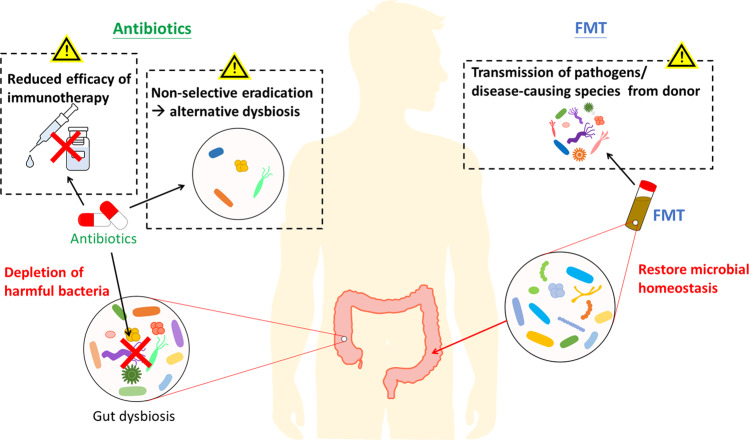
Putative mechanisms of action of antibiotics and fecal microbiota transplantation (FMT) and their associated safety concerns. Gut dysbiosis often leads to the development of various diseases, therefore antibiotics and fecal microbiota transplantation are viable approaches to reverse dysbiosis and restore homeostasis. Antibiotics are effective in eradicating the pathogenic or harmful bacteria, but its non-selective antimicrobial actions may lead to another state of dysbiosis by killing the commensal microflora. It may also compromise the efficacy of cancer immunotherapy, which anticancer activity is modulated by commensal microbiota. On the other hand, FMT introduces a new bacterial community to the recipient, aiming to reverse the established dysbiosis. However, owing to the many unknown components presented in the donor’s samples, it also carries the risk of transmitting pathogens or disease-causing genes to the recipient.

Aberration of the intestinal microbial community has been linked with impaired gut barrier function, inflammation, and eventually carcinogenesis and tumor progression. Antibiotics treatment, as to deplete gut microbiome and reverse the detrimental dysbiosis, thus becomes a rational investigational approach for cancer prevention and therapy (Fig. 3). Usually administered by gavage or drinking water, antibiotics are commonly used in in vivo models to study the impacts of gut microbiome on cancer or other inflammatory diseases. Indeed, antibiotic-mediated microbiome depletion was reported to attenuate CRC development in various studies [82–84], and such protective effect is suggested to be primarily through the elimination of the carcinogenic *Bacteroides fragilis* [85], as well as bacteria that are associated with mucin degradation [86], inflammation and DNA methylation [87].

Antibiotic treatment is also implicated in suppressing tumor proliferation, invasion and growth. In mice bearing CRC xenograft, treatment with metronidazole eradicates *Fusobacterium* colonization, and reduces CRC proliferation [88], suggesting antibiotics as a potential intervention for *Fusobacterium*-enriched CRC patients. Another study that investigated in the role of neutrophils in colon tumors has reported a distinct microbiota composition in mice with or without neutrophil depletion, whereas antibiotics treatment reduces bacterial load in tumor and inhibits tumor invasion [89]. Meanwhile, antibiotics treatment is even suggested as an immunotherapeutic strategy, as gut microbiome depletion by antibiotics was shown to elicit antitumor immune response and suppresses tumor growth in metastatic mouse models [90].

However, antibiotic administration, being the most aggressive means to manipulate gut microbiota composition, has been controversial in its role in cancer management. Although gut microbiome depletion was shown to inhibit cancer progression, accumulating evidence has told another side of the story that antibiotics can compromise immunotherapy efficacy or induce disease progression by creating further microbial dysbiosis (Fig. 3).

#### Compromise of immunotherapy efficacy

The pharmacological principles of immunotherapy pertain to the manipulation of innate immunity and subsequent activation of antitumor immune response. Hence, tumor microenvironment is a critical factor that conditions the therapeutic outcomes. The gut microbiome, by interfering with host immunity, has played an indispensable role in treatment response. That being said, the non-selective eradication of these commensal bacteria by antibiotics can abrogate the antitumor immunity.

Several studies have pointed out the involvement of some specific gut bacteria, such as *Bacteroides thetaiotaomicron*, *Bacteroides fragilis* [91], *Bifidobacterium* species [92], *Akkermansia muciniphila* [93], *Alistipes shaii* [94], in response to immunotherapy. As a consequence, depletion of microbiota using antibiotics impairs the efficacy and results in treatment resistance. For instance, Vétizou et al. demonstrated that an antibiotic cocktail consisting ampicillin, colistin, and streptomycin, or imipenem alone, abolished the cytotoxic T-lymphocyte-associated antigen 4 (CTLA-4) blockade and restored tumor progression in sarcoma, melanoma and CRC mouse models [91]. In the meantime, antibiotic-mediated microbiota depletion may also exacerbate treatment toxicity, which in clinical settings, leads to discontinuation or dose reduction. Recent study has revealed that the role of Bifidobacterium in mitigating autoimmune toxicities without compromising treatment efficacy, whereas vancomycin pre-treatment to mice with colitis and treated with anti-CTLA-4 therapy results in more severe and fatal manifestation of colonic inflammation [95].

Clinical observations have been consistent with these preclinical findings. Despite the lack of prospective trials, a retrospective study has reported that concomitant use of antibiotics and immunotherapy is associated with a high risk of disease progression, as well as shorter progression-free survival (PFS) and overall survival (OS) [96]. Similarly, in patients with antibiotic exposure 30 days prior to immunotherapy initiation, they also have a higher tendency of experiencing primary resistance and generally a shorter survival [97], which is consistent with the findings of another study that antibiotic use is a predictor of resistance toward programmed cell death protein 1 (PD1)-based immunotherapy [93].

#### Induction or exacerbation of dysbiosis

In animal models, antibiotic treatment is a common strategy employed to eradicate carcinogenic bacteria, and by doing so, antibiotic administration is often reported to protect against cancer development or attenuate tumor proliferation in these studies [82, 89]. Clinical studies, however, have reported disparate findings that antibiotic use is closely associated with increased risk of CRC development, instead of a protective effect [98, 99]. In fact, such findings are biologically plausible – as antibiotic treatment is a non-selective means of depletion, it can easily exacerbate or create another state of dysbiosis, including but not limited to reduced microbiome diversity, altered abundance of specific species or taxa and increased susceptibility to invading pathogens [100]. In a long-term prospective cohort study, it was further discovered that exposure to antibiotics during early to middle adulthood, but not recent antibiotic use (within the past 4 years), increased risk of CRC development [101]. This suggests that antibiotic-mediated dysbiosis is probably a long-term problem that persists even after treatment cessation, and may not be easily reversed or rectified.

Studies regarding antibiotic use and cancer risk are still ongoing, yet are often limited by the nature of observational studies. In the clinical setting, it is commonly complicated by confounding factors that may be difficult to be ruled out, one of which is the issue of “confounding by indications” [102]. There might not a causal linkage between antibiotic use and cancer risk, but antibiotics are prescribed for an underlying medical condition associated with CRC. For example, as addressed by Dik et al. patients with immune deficiency may be more susceptible to cancers but also bacterial infection that requires antibiotic treatment [99]. Alternatively, these patients may be colonized with a specific pathogen, which is carcinogenic but concomitantly induces inflammation that necessitates antibiotic use. To examine the true effects of antibiotic exposure to CRC development, such distortion from confounders should be carefully evaluated and addressed in further studies.

### Fecal microbiota transplantation

#### Reversion of established microbial dysbiosis

With the increasing understanding of how altered gut microbiota impacts on diseases, fecal microbiota transplantation (FMT) becomes an emerging biotherapeutic in recent years. By administrating fecal transplants from healthy donors to patients’ gastrointestinal tract, FMT introduces a healthy, disease-free microbial population to a dysbiotic community, which then restores microbial homeostasis (Fig. 3) and may be potentially useful in ameliorating various gastrointestinal disorders, including IBD, irritable bowel syndrome and *Clostridium difficile* infection (CDI) [103]. Compared with other modulating strategies, FMT seems to confer several advantages over the others. While it increases microbial diversity and does not result in disruption of microbial gut ecology as in antibiotic treatment, its long-term engraftment also allows it to be designed as a single-dose regimen, thereby conferring therapeutic benefits over probiotics and prebiotics, whose colonization appears to be transient [104].

Currently, experimental evidence regarding FMT efficacy mostly concentrates on CDI treatment, while its application in other gastrointestinal disorders, especially in CRC, is highly unexplored. A recent study has reported that fecal transplants from wild mice to laboratory mice has improved host fitness and resistance against dextran sodium sulfate (DSS)/ azoxymethane (AOM)-induced colorectal tumorigenesis [105]. Introducing a healthy microbiome, which in this study refers to the wild mice’s natural microbiota exposed to different immune stimuli instead of restrictive environment in laboratory, is thereby suggested to alter microbiota composition and exert protective effect against CRC development.

Yet, despite the enticing pilot data, numerous uncertainties regarding clinical FMT are yet to be answered, particularly in its safety profile. Preliminary preclinical and clinical studies have suggested some potential risks associated with FMT in clinical use (Fig. 3). Although they are not supported by solid evidence and currently remains inconclusive, clinicians should stay skeptical and cautious about them.

#### Transmission of unrecognized pathogens

Owing to the rapid introduction of FMT into practice and the lack of large-scale prospective trials, current safety evidence regarding FMT intervention has been limited. In short term, FMT is considered a safe intervention. Some patients receiving FMT do develop adverse events such as constipation, diarrhea, belching, abdominal distension, but these side effects are usually transient and subside a few days after transplantation [106]. Yet, what has evoked the controversies is the recent release of safety alert from the Food and Drug Administration (FDA) [107], warning on the potential risks of transmitting multi-drug resistant bacteria and developing subsequent life-threatening infections. In the case reported by FDA, two immunocompromised patients are infected by extended-spectrum beta-lactamase (ESBL)-producing Escherichia coli, which are later isolated in donor’s stool preparations, and one died from that infection episode.

Several cases reports also documented the infection events subsequent to FMT, including norovirus gastroenteritis [108], *Escherichia coli* bacteremia [109] and cytomegalovirus infection [110]. It, however, remains difficult to draw a confident conclusion of whether there is a causative relationship between FMT and these infection episodes – some infections are speculated to stem from operating personnel and community exposures [108]. Yet the indisputable truth is that unrecognized infectious agents present in the fecal transplants do pose deleterious risks on FMT recipients, thereby necessitating a more stringent protocol for donor screening.

#### Dissemination of disease-causing genes

On top of transmitting unrecognized pathogens, another understudied area is the potential risk of disseminating disease-causing genes. The gut microbiota has been known to be associated with various human diseases, including gastrointestinal diseases, obesity, autism, cardiovascular disorders and autoimmune disorders [111]. During the process of FMT, it carries the possibility that some unknown components in donor’s stool preparations can be passed on to recipients, causing alteration of recipient’s microbiota and consequently induction of chronic diseases. Preclinical studies have shown that transplanting human feces from obese individuals to germ-free mice fed with low-fat diet induces obesity as well as obesity-related metabolic phenotypes [112]. There is also one case report that a woman developed obesity after receiving FMT intervention from a healthy but overweight donor [113]. Apart from obesity, atherosclerosis is also reported to be “transmissible” from donors to recipients, which is mechanistically through altered production of the pro-atherogenic trimethylamine-N-oxide (TMAO) [114]. In a gnotobiotic mouse model, fecal transplantation is capable of transferring cutC gene, which is involved in encoding choline TMA-lyases and subsequent production of TMAO from its precursors. As a result, the increased TMA-lyase activity leads to increased plasma TMAO levels and heightened thrombotic potential in recipient mice [115]. Although it remains a theoretical risk, these studies have raised a legitimate concern and alerted us on potential complications associated with FMT. To date, clinical evidence is still lacking, and long-term clinical follow-ups are warranted to confirm the causality.

## The wider health implications in CRC

With the increasing understanding of how gut microbiota impacts on host health as well as their mechanisms, manipulation of the gut microbiome may be a novel strategy for cancer prevention and treatment. Currently, gut microbiota modulation, mostly by using probiotics, is suggested to exert three distinct benefits to CRC patients or high-risk individuals through preventing CRC incidence, alleviating treatment-related side effects and potentiating efficacy of anticancer therapy.

### CRC prevention

Cancer prevention is the most early researched area that attempts to integrate gut microbiota manipulation in clinical oncology. Using probiotics, prebiotics or synbiotics (referring to the combination of the former two to achieve synergism), various studies have reported a protective effect in CRC mice models such as DMH or AOM models, as reviewed elsewhere [116]. Functioning in a species- and strain-specific manner, some probiotics reduced tumor incidence, tumor size and tumor number, or prevented precancerous lesions (aberrant crypt foci). The effect on CRC prevention can be generally attributed to several mechanisms, including suppressing inflammation [117–119], enhancing apoptosis of early tumor cells [117, 118], restoring gut barrier function and correcting microbiota composition [119].

Two randomized-controlled trials have evaluated role of probiotics and prebiotics in CRC prevention [120, 121] (Table 1). Consistent with the in vitro findings, administration of selected probiotic strains and dietary fiber has shown to downregulate inflammation (as evidenced by the prevention of interleukin-2 increase) and reduce genotoxin exposure, which are both plausible mechanisms for CRC protection [120]. However, despite the alteration of some CRC biomarkers and prevention of tumor atypia, results from both trials did not indicate strong evidence of CRC prevention, as ultimately the tumor occurrence rate does not differ significantly between treatment and non-treatment group [121]. Further large-scale long-term clinical trials are needed to confirm such protective effects in clinical settings.

**Table 1:** Randomized-controlled trials of gut microbiota modulation in CRC prevention.

Patient population	*n*	Intervention	Duration	Status	Results	Ref.
CRC-resected or polypectomized patients	80	Synbiotic combination (oligofructose-enriched inulin + Lactobacillus rhamnosus GG and Bifidobacterium lactis Bb12)	12 weeks	C	• Polypectomized patients: reduced exposure to genotoxins, prevented IL-2 increase • CRC patients: increased IFN-γ production	[123]
Patients who are currently free of tumor with at least 2 colorectal tumors removed.	380	(1) wheat bran (2) Lactobacillus casei (3) wheat bran + Lactobacillus casei (4) Control	4 years	C	• Wheat bran alone: increased number of large tumors after 4 years, adjusted OR 1.57 (95% CI 1.04–2.37) • L. casei group: reduced occurrence of tumors with moderate or severe atypia, adjusted OR 0.65 (95% CI 0.43–0.98) • Combination group: no synergistic effect observed	[124]

*C* completed, *CI* confidence interval, *IFN-γ* interferon-γ, *IL-2* interleukin-2, *OR* odds ratio.

### Alleviate treatment-related side effects

Chemotherapy and radiotherapy are commonly employed in CRC treatment, yet their toxicities often prevent further dose escalation or lead to treatment discontinuation. Gastrointestinal mucositis is one of the most well-documented side effects, which is characterized by weight loss, diarrhea, shortening of villi, intestinal inflammation and damage to intestinal integrity [122]. By directly altering the colonic environment, manipulating the gut microbiota is therefore hypothesized to mitigate the side effects. Various studies have shown that several probiotics strains, or their supernatant, can ameliorate chemotherapy-induced mucositis, as observed by reduced incidence of diarrhea and weight loss, primarily through suppressing inflammation [123–125], restoring gut barrier integrity [126] and inhibiting intrinsic apoptosis [125]. Dietary prebiotic fiber was also found to exert beneficial effects in relieving irinotecan toxicity, accompanied by a strong correlation with increased butyrate production [127]. Meanwhile, FMT from healthy mice to chemotherapy-treated or irradiated mice also yields promising results. By restoring gut microbiota homeostasis, FMT is shown to effectively protect mice from treatment-related gastrointestinal toxicity and improve animal survival rates [128, 129].

Myelosuppression is another important dose-limiting toxicity for many chemotherapeutic agents. One study has attempted to incorporate probiotic treatment into chemotherapy and evaluate its efficacy to protect against myelosuppression in mice models. Two probiotic strains, *Lactobacillus casei* CRL431 and *Lactobacillus rhamnosus* CRL1506, are found to foster recovery of myeloid cells and neutrophils after cyclophosphamide treatment, facilitate phagocytosis in infection sites and protect mice from opportunistic infection with *Candida albicans* [130]. Although the molecular mechanism of such protective effect remains unclear, this study has opened a new research direction for the clinical implications of probiotics.

In view of the preclinical findings, several clinical trials have evaluated the use of probiotics in CRC patients to alleviate treatment-induced gastrointestinal side effects (Table 2). These studies can be roughly classified in accordance with their clinical settings, namely during chemotherapy or radiotherapy, preoperative and postoperative management. Most of these studies have reported positive results for probiotic use in CRC management, including but not limited to reduced incidence of diarrhea [131–136] and infectious complications [136–138], improved recovery of bowel movement [134, 135], enhanced gut barrier integrity [136, 137] and reduced inflammation [139]. A study has also evaluated the use of guar gum, a potential prebiotic, in CRC patients receiving 5-FU-based chemotherapy, but such fiber does not seem to improve patient tolerability to chemotherapy [132].

**Table 2:** Randomized-controlled trials of gut microbiota modulation in alleviating treatment-related side effects.

Patient population	*n*	Intervention	Duration	Status	Results (completed trials)/ measured outcomes (ongoing trials)	Clinical trial registration number	Ref.
During chemotherapy							
CRC patients starting new irinotecan-based chemotherapy	46	Colon Dophilus (containing 10 probiotic strains) vs placebo	12 weeks	C	Reduced incidence of overall diarrhea, grade 3-4 diarrhea and enterocolitis, less use of antidiarrheal drugs in probiotic group	NCT01410955	[134]
Patients with Dukes’ B or C CRC or metastatic CRC without overt metastases (Dukes’ D)	150	Adjuvant 5-FU based chemotherapy (Mayo or simplified de Gramont regimen with or without Lactobacillus rhamnosus GG and guar gum	24 weeks	C	Reduced incidence of grade 3-4 diarrhea, less abdominal discomfort, less cases of chemotherapy dose reduction in Lactobacillus group; no difference in treatment tolerability in guar gum group	/	[135]
Metastatic CRC patients receiving FOLFIRI regimen	76	Probiotic (Omni-Biotic 10) vs placebo vs loperamide	160 days (2 full chemotherapy cycles)	O	- Incidence of grade III/IV diarrhea - Concentration of zonulin and vitamin D - Quality of life	NCT03705442	/
During radiotherapy							
Patients with sigmoid, rectal or cervical cancers who received adjuvant postoperative radiation therapy	239	VSL#3 vs placebo	Concomitant with radiotherapy	C	Less incidence of overall diarrhea, grade 3-4 diarrhea, less bowel movements, longer time to use loperamide from the start of study in probiotic group	/	[136]
Patients with gynecologic, rectal, or prostate cancers	229	Bifilact (Lactobacillus acidophilus LAC-361, Bifidobacterium longum BB-536) vs placebo	Concomitant with radiotherapy	C	Less patients with moderate or severe diarrhea after 60 days of treatment, less bowel movement and abdominal pain in probiotic group	NCT01839721	[137]
Patients with stage I-III CRC	40	Probiotic capsule containing 7 Lactobacillus and 5 Bifidobacterium species vs no treatment	Concomitant with radiotherapy	O	- Level of immunoglobulin (IgA, IgG, IgM), inflammatory cytokines (IL-1, IL-6, IL-10) - Quality of life - Gastrointestinal toxicity	NCT03742596	/
Perioperative							
Patients with sporadic CRC	60	Probiotic mixture (Bifidobacterium longum, Lactobacillus acidophilus, Enterococcus faecalis) vs placebo	5 days preoperatively and 7 days postoperatively	C	Lower incidence of diarrhea, less days to first flatus and defecation in probiotic group	ChiCTR-TRC-13003332	[138]
CRC patients scheduled to undergo radical colorectomy	114	Probiotic mixture (Lactobacillus plantarum CGMCC No. 1258, Lactobacillus acidophilus LA-11, Bifidobacterium longum BL-88) vs placebo	6 days preoperatively and 10 days postoperatively	C	Reduced transepithelial permeability, reduced bacterial translocation, increased tight junction protein expression, improved recovery of peristalsis, lower incidence of diarrhea and infectious complications in probiotic group	ChiCTR‐TRC‐00000423	[139]
CRC patients scheduled to undergo radical colorectomy	138	Probiotic mixture (Lactobacillus plantarum CGMCC No. 1258, Lactobacillus acidophilus LA-11, Bifidobacterium longum BL-88) vs placebo	6 days preoperatively and 10 days postoperatively	C	Decreased serum zonulin concentration, reduced infection rate, infectious complications, duration of pyrexia and duration of antibiotic therapy in probiotic group	ChiCTR‐TRC‐00000423	[140]
Postoperative							
CRC patients scheduled for colorectal resection	164	Probiotic mixture (Lactobacillus acidophilus LA-5), Lactobacillus plantarum, Bifidobacterium lactis BB-12, Saccharomyces boulardii) vs placebo	30 days after surgery	C	Reduced incidence of postoperative complications (pneumonia, infections and anastomotic leakage) in probiotic group	NCT02313519	[141]
CRC patients scheduled for colorectal resection	52	HEXBIO (containing 6 viable probiotic strains) vs placebo	Start 4 weeks after surgery, continue for 6 months	C	Reduced level of proinflammatory cytokines in probiotic group; no difference in diarrhea severity scores	NCT03782428	[142]

*C* completed, *O* ongoing.

However, despite the preliminary clinical benefits demonstrated in these short-term studies, there is a lack of studies reporting the impact of probiotics on clinical outcomes, such as progression-free survival (PFS) and overall survival (OS). Whether these clinical benefits be translated to improvement of long-term outcomes remains unknown to clinicians.

### Potentiate efficacy of anticancer therapy

In recent years, increasing interest is drawn to the potential role of gut microbiota in augmenting therapeutic efficacy of anticancer drugs. Although currently most studies are restrained to preclinical models, some promising data is reported, suggesting another possible clinical implication of gut microbiota manipulation.

Modulating the gut microbiota composition is a potential strategy to improve tumor response to chemotherapeutic agents. Over a decade ago, there were some attempts of adding dietary prebiotic fiber into anticancer treatment. The study demonstrated that supplementing diet rich in inulin or oligofructose inhibits growth of transplantable tumor in mice and potentiated efficacy of 6 different cytotoxic drugs at their subtherapeutic doses. The precise mechanism was not elucidated in that study but was hypothetically mediated by the prebiotic properties of inulin and oligofructose [140]. Meanwhile, gut microbiota depletion using antibiotics was shown to confer clinical benefits to CRC patients by overcoming chemotherapeutic resistance. The gut microbiota, specifically the intratumor bacteria, was found to induce gemcitabine resistance through enzymatic inactivation of the drug, while a gemcitabine-ciprofloxacin combination therapy abrogates resistance and potentiate treatment efficacy [141].

The gut microbiota is also known to influence chemotherapy and/or immunotherapy efficacy by modulating immunity. Cyclophosphamide, which possesses functions of both chemotherapy (as alkylating agent) and immunotherapy (by stimulating antitumor immune response), was shown to cause translocation of certain species of Gram-positive bacteria (*Lactobacillus johnsonii, Lactobacillus murinus, Enterococcus hirae*) into secondary lymphoid organs. Such translocation appeared to be essential for eliciting antitumor “pathogenic” Th17 cells and memory Th1 immune response, as antibiotic-treated mice failed to produce the response and conferred resistance to cyclophosphamide [142]. Gavage treatment with *Enterococcus hirae* and *Barnesiella intestinihominis*, two proposed probiotics, has restored the drug response in antibiotic-treated mice [143].

On the other hand, immunotherapy efficacy appears to be heavily influenced by gut microbiota composition. Oral administration of probiotics, such as *Bifidobacterium* species [92] and *Akkermansia muciniphila* [93], or FMT [144] from treatment-responsive patients, substantially enhanced the PD1-based immunotherapy and abolished tumor outgrowth, mechanistically through the augmented dendritic cell and T cell response [92]. Although these studies are not employing CRC models, understanding how gut microbiota modulates immune response may be critical to facilitate positive therapeutic outcomes in CRC patients receiving immunotherapy, or even to overcome resistance harbored by non-responders.

To our best knowledge, no clinical trials evaluating gut microbiota manipulation and treatment efficacy are published currently. A few clinical trials are initiated and now at the recruiting stage (Table 3). It remains obscure whether these preclinical findings can be successfully translated to clinical application.

**Table 3:** Ongoing clinical trials of gut microbiota modulation in potentiating efficacy of anticancer therapies.

Patient population	*n*	Intervention	Primary outcomes	Secondary outcome	Location	Status	Clinical trial registration number
Chemotherapy							
Patients with metastatic CRC	50	Chemotherapy + Weileshu (Lactobacillus salivarius AP-32, Lactobacillus johnsonii MH-68) vs chemotherapy alone	PFS	OS	Zhejiang, China	Not yet recruiting	NCT04021589
Patients with metastatic CRC	140	Chemotherapy + targeted therapy + Bifico (Lactobacillus acidophilus and Bifidobacterium) vs chemotherapy + targeted therapy	ORR	/	Zhejiang, China	Not yet recruiting	NCT04131803
Rectal cancer patients receiving concurrent chemotherapy and pelvic Radiation therapy	160	VSL#3 vs placebo	Impact of probiotics to increase tumor regression grade (TRG) 1-2 rate	- Acute bowel toxicity - Pathological complete response - Sphincter saving surgery - Disease-free survival - Late toxicity (at 12-36 months)	Rome, Italy	Recruiting	NCT01579591
Immunotherapy							
Melanoma patients resistant/ refractory to PD-1 therapy	20	Single-arm: FMT from anti-PD1 responders through colonoscopy + PD-1 therapy	ORR	- T cell composition - Immune profile - T cell function	Pennsylvania, United States	Recruiting	NCT03341143
Patients with solid tumors (including non small cell lung cancer, renal cell carcinoma, bladder cancer or melanoma).	132	Single-arm: MRx0518 + Pembrolizumab	Adverse events	- Clinical benefits (ORR, DOR, DCR, PFS) - Tumor biomarkers - Microbiome composition - OS	Texas, United States	Recruiting	NCT03637803

*PFS* progression-free survival, *OS* overall survival, *ORR* objective response rate, *DOR* duration of response, *DCR* disease control rate.

## Conclusion and perspective

Technological advances in taxonomic profiling have made a breakthrough in microbiome research regarding cancer pathophysiology. Accumulating preclinical evidence has suggested gut microbiota manipulation as a potential therapeutic strategy for prevention and treatment of cancer. However, before translating to bedside application, some fundamental questions are yet to be answered.

Firstly, what is defined as an “abnormal” microbiome that necessitates therapeutic interventions? At present, no quantitative definitions regarding microbial dysbiosis are available, as this concept seems to be host-specific and disease-specific [145]. Therefore, before making a clinical decision of initiating an intervention, a clear definition and precise patient selection criteria is critical – especially when we acknowledge that those manipulating strategies do carry variable risks. The second question that ought to be answered is the prerequisite for effective intervention. Increasing studies have revealed that not all subjects respond equally to gut microbiota modulating treatment, but it highly depends on the baseline characteristics, including genetic background [70], gut barrier function [146] and microbiome diversity [147]. Development of personalized microbiome therapy, thus, is the key to successful clinical treatment. Lastly, data regarding human clinical trials remains sparse. Clinicians must be cautious about it and should not arbitrarily extrapolate animal data to clinical application, as cross-species translation can be potentially dangerous – the representative example will be antibiotics, which often demonstrate promising animal results but is shown to create numerous problems in clinical settings.

Despite the many unknowns, we believe that gut microbiota modulation has the potential that deserves further investigation of its role in prevention and treatment of colon cancer. With continuous efforts in preclinical and clinical studies, we will be eager to see how it can be translated into clinical practice and provide additional therapeutic aids to high-risk individuals and patients.
